# Low-Frequency TMS Results in Condition-Related Dynamic Activation Changes of Stimulated and Contralateral Inferior Parietal Lobule

**DOI:** 10.3389/fnhum.2021.684367

**Published:** 2021-07-23

**Authors:** Janine Jargow, Katharina Zwosta, Franziska M. Korb, Hannes Ruge, Uta Wolfensteller

**Affiliations:** Faculty of Psychology, Technische Universität Dresden, Dresden, Germany

**Keywords:** fronto-parietal control network, default mode network, functional magnetic resonance imaging, inferior parietal lobe, offline TMS, functional reorganization, intra-network compensation

## Abstract

Non-invasive brain stimulation is a promising approach to study the causal relationship between brain function and behavior. However, it is difficult to interpret behavioral null results as dynamic brain network changes have the potential to prevent stimulation from affecting behavior, ultimately compensating for the stimulation. The present study investigated local and remote changes in brain activity via functional magnetic resonance imaging (fMRI) after offline disruption of the inferior parietal lobule (IPL) or the vertex in human participants via 1 Hz repetitive transcranial magnetic stimulation (rTMS). Since the IPL acts as a multimodal hub of several networks, we implemented two experimental conditions in order to robustly engage task-positive networks, such as the fronto-parietal control network (on-task condition) and the default mode network (off-task condition). The condition-dependent neural after-effects following rTMS applied to the IPL were dynamic in affecting post-rTMS BOLD activity depending on the exact time-window. More specifically, we found that 1 Hz rTMS applied to the right IPL led to a delayed activity increase in both, the stimulated and the contralateral IPL, as well as in other brain regions of a task-positive network. This was markedly more pronounced in the on-task condition suggesting a condition-related delayed upregulation. Thus together, our results revealed a dynamic compensatory reorganization including upregulation and intra-network compensation which may explain mixed findings after low-frequency offline TMS.

## Introduction

Over the past decades, a plethora of studies have investigated behavioral changes after transcranial magnetic stimulation (TMS, for reviews see e.g., Pascual-Leone et al., 2000; Rushworth and Taylor, 2006; Koch and Rothwell, 2009; Rossini et al., 2010; Crivelli and Balconi, 2017; Klaus and Schutter, 2018). Although brain stimulation is a promising approach to study the causal relationship and close the explanatory gap between brain function and behavior, it has its drawbacks as exemplified in interpreting behavioral null results after stimulation (e.g., Rossi et al., 2006; Zanto et al., 2013; Gohil et al., 2016; Bor et al., 2017; Engelen et al., 2018; Layher et al., 2018; Lopez-Alonso et al., 2018; Codol et al., 2020; see also De Graaf and Sack, 2011). There are a number of possible explanations for TMS null results ranging from the stimulated brain region not being causally involved in the tested behavior (Rossi et al., 2006; Gohil et al., 2016) to dynamic brain network changes compensating for the stimulation (Lee et al., 2003; O’Shea et al., 2007; Zanto et al., 2013; Hartwigsen, 2018). The present paper focuses on stimulation-induced dynamic changes in brain activity that may constitute compensatory mechanisms (Ruff et al., 2006; Sack et al., 2007; Hartwigsen et al., 2017).

Compensatory reorganization, i.e., altered activity and connectivity patterns (Hartwigsen, 2018) after stimulation can be readily investigated with functional magnetic resonance imaging (fMRI). Combining brain stimulation with brain imaging (e.g., Siebner et al., 2009; Bergmann et al., 2016; Hartwigsen et al., 2017; Beynel et al., 2020; Castrillon et al., 2020) can thereby help to explain differences in the modulatory effects of brain stimulation on behavior. For instance, stimulation effects seem to depend not only on stimulation frequency, as previously suggested (Chen et al., 1997; Pascual-Leone et al., 1998; Boroojerdi et al., 2000; Nyffeler et al., 2006; Speer et al., 2009). In fact, the heuristic that low-frequency rTMS generally inhibits cortical excitability (Pascual-Leone et al., 1998) is not undebated (Beynel et al., 2020) in light of studies combining rTMS and fMRI that reported (compensatory) excitatory after-effects after low-frequency stimulation on remote brain areas (Lee et al., 2003; O’Shea et al., 2007; Beynel et al., 2020; Castrillon et al., 2020). Stimulation effects seem to depend on several further factors e.g., the stimulated brain region (Castrillon et al., 2020), the post-stimulation task or condition (Lee et al., 2003; O’Shea et al., 2007) and the time passed after stimulation (O’Shea et al., 2007).

More specifically, Lee et al. (2003) reported decreased connectivity of the stimulated motor cortex and an additional movement-related activity increase in the contralateral premotor cortex after 1 Hz rTMS. This hints at a complex reorganization which depends on the post-stimulation state of the brain region under investigation (i.e., whether it is involved in task performance or not).^1^ Furthermore, O’Shea et al. (2007) have shown that action selection after 1 Hz rTMS over left dorsal premotor cortex (PMd) was impaired only for a short period after rTMS, corresponding to roughly a third of the stimulation time. Interestingly, 5 min after rTMS, the right PMd showed increased activation suggesting compensation by the contralateral brain area. This interpretation was supported by a second experiment revealing enduring performance disruption after bilateral PMd stimulation. These results showcase the importance of timing of behavioral modulation and compensatory reorganization after rTMS. In accordance, other studies have reported behavioral after effects of low-frequency rTMS to have a duration half as long as the stimulation train (Robertson et al., 2003; Eisenegger et al., 2008). Specifically, Eisenegger et al. (2008) found that brain activation changes following 15 min of 1 Hz rTMS returned to baseline after 9 min. Based on these findings, it is conceivable that the neural and behavioral effects of rTMS change in the course of an experiment if its duration is approximately as long as the stimulation period. To sum up, the effects of low-frequency rTMS seem to depend on the post-stimulation condition of the stimulated area and seem to change already during the first minutes after stimulation. Therefore, the present study aims at investigating after-effects and rapid reorganization following offline rTMS in terms of local and remote changes in brain activity, taking into consideration both condition-dependence and timing aspects.

As the target site of stimulation we chose the angular gyrus (AG) – a multimodal region within the IPL (Rademacher et al., 1992) which is considered as a main hub of several brain networks including the default mode network (DMN; Buckner et al., 2008; Hagmann et al., 2008; Igelström and Graziano, 2017) and the fronto-parietal control network (Vincent et al., 2008; Igelström and Graziano, 2017; Dixon et al., 2018). Therefore, we employed two different experimental conditions, i.e., an on-task and an off-task condition (for a similar on-task, off-task design see Kam et al., 2013; Turnbull et al., 2019; Riemer et al., 2020). The on-task condition was chosen to robustly engage task-positive networks, such as the fronto-parietal control network. To achieve this, we used a modified spatial Simon task (Simon and Wolf, 1963) with novel stimulus-response rules for each task block. The off-task condition was chosen to robustly engage the task-negative DMN. Relying on the notion that the DMN is activated when no external task is presented (Buckner et al., 2008), during off-task blocks participants had to merely fixate a target cross.

Repetitive low-frequency (1 Hz) stimulation was administered to the right AG or the vertex for 20 min directly before measuring fMRI during alternating blocks of on-task and off-task conditions. Thereby, we were able to investigate condition-related and condition-unrelated effects on rapid brain activity reorganization after rTMS. Based on the reviewed literature, we hypothesized potentially compensatory reorganization following stimulation to take place depending on whether the current condition demanded it. Furthermore, in order to test time-dependent rTMS after-effects, we compared activation changes in the early and late phase of the 8 min fMRI session following the rTMS stimulation at AG or vertex, respectively. If present, condition-related reorganization should be fully visible in the late phase of the fMRI session following rTMS of the AG, but not the vertex.

## Materials and Methods

### Participants

A total of 22 participants (10 male) were recruited and screened for suitability for TMS (Rossi et al., 2011). This sample size was determined based on comparable studies (Esslinger et al., 2014; Min et al., 2016; Peschke et al., 2016; Battelli et al., 2017; Klaus and Schutter, 2018). The final sample comprised 20 participants (9 male, mean age = 25, SD = 3.31, range 19 – 33). Two additional participants were excluded from the analysis, one due to an incidental neurological finding and the other one due to insufficient performance in the on-task condition (error rates > 3 SDs above session mean). All participants were right-handed (Edinburgh Handedness Inventory, Oldfield, 1971) and had normal or corrected-to-normal vision, including normal color vision.

Stimulation order was balanced across participants. One group (*n* = 10) received vertex stimulation first (4 male, mean age: 24.5, mean motor threshold: 49.4% of the maximum stimulator output), while the other group (*n* = 10) received AG stimulation first (5 male, mean age: 25.5, mean motor threshold: 48.9% of the maximum stimulator output). The experimental protocol was approved by the Ethics Committee of the Technische Universität Dresden (IORG0001076/IRB00001473). Participants were instructed, gave written informed consent and were randomly assigned to one of the two groups either starting with vertex or with AG rTMS. They received financial compensation of 24 € for their participation and were thanked and debriefed at the end of the experiment.

### Experimental Procedure

**FIGURE 1:**
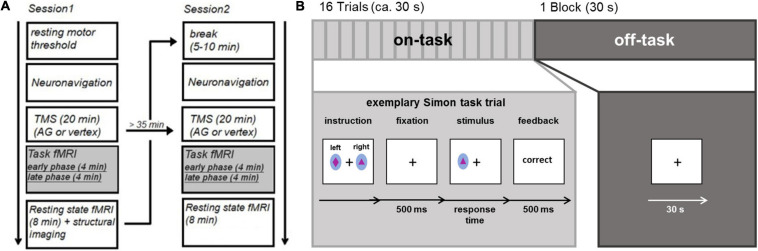
**(A)** Experimental procedure **(B)** task fMRI consisting of external attention condition (Simon task) and internal attention condition.

Prior to the experiment participants performed a practice run to familiarize them with the experimental task. The study consisted of two stimulation sessions (see Figure 1A), separated by at least 35 min. The individual resting motor threshold was determined prior to the first stimulation. In each session, we stimulated the vertex or the AG followed by the acquisition of fMRI data. Scanning started 3–5 min (mean delay: 3 min and 50 s, SD: 36.8 s) after rTMS. Each fMRI session contained eight on-task blocks and eight off-task blocks. An extended resting state block started 12–15 min after stimulation. However, we refrain from discussing this resting state result in the main paper, as the results may be influenced by prior task execution and therefore hard to interpret (the interested reader is referred to the Supplementary Material). The second stimulation session differed from the first regarding the stimulated brain region (AG or vertex), but was otherwise identical.

### Experimental Conditions

During the fMRI session, participants alternated between performing 8 blocks consisting of 16 trials of a spatial Simon task (Simon and Wolf, 1963; Simon, 1969) and 8 fixation blocks (30 s), thus essentially alternating between on-task and off-task blocks. At the beginning of each on-task block, subjects were presented with novel stimulus-response rules. Two stimuli were shown and subjects were instructed to respond to one stimulus with the left key and to another stimulus with the right key (see Figure 1B) as soon as they detected the stimulus. For each block, a new pair of colored stimuli (ellipsoids filled with different geometrical forms subtending visual angles of 0.65° in width and 0.81° in length) was used. Stimuli were displayed on a back-projection screen, which could be seen via a mirror attached to the MRI head coil. Each trial started with a fixation cross displayed at the center of the screen for 500 ms. Next, the stimulus was presented for 700 ms (or until a response was made). The stimuli were randomly positioned to the left or right of a centrally presented fixation cross (distance: 1.2°). Participants had to respond within a time window of 1,000 ms after stimulus onset with a left or right button press followed by performance feedback which was presented for 500 ms. Stimulus location and the required response were either spatially compatible (i.e., on the same side) or incompatible (i.e., on opposite sides). As feedback, the German words for “correct,” “wrong,” and “too slow” were presented in the center of the screen; “wrong” and “too slow” were presented in red ink. The inter-trial interval following the feedback had a maximum duration of 2,000 ms and varied depending on RT. During off-task blocks, a fixation cross was presented in the center of the screen for 30 s. The experiment was controlled by E-Prime 2.0.

### Stimulation Procedure

TMS was carried out using a MagPro X100 with Magoption (MagVenture GmbH, Willich, Germany) and a MagVenture figure of eight MCF-B65 coil (75-mm diameter double-circle). Before stimulation, we determined the individual resting motor threshold (Rossini et al., 1994, 2015), i.e., the minimum percentage of the stimulator output required to elicit a motor response. After locating primary motor cortex, the stimulation intensity was decreased until 5 out of 10 pulses resulted in an observable twitch of the index finger muscle (abductor pollicis brevis). This stimulation intensity was taken as the individual resting motor threshold.

Participants’ anatomical T1-weighted MRI brain images (acquired during previous studies) were used to guide stimulation via PowerMAG View Navigation software (Mag & More, Munich, Germany). Neuronavigation was conducted using tracking devices and an infrared camera (Polaris Vicra; Northern Digital Inc., ON, Canada). First, the individual structural brain images were co-registered to each participant’s head. In one session, the AG coordinate (45 −58 33), derived from a previous study (Zwosta et al., 2015), was projected onto individual brain space and targeted for stimulation using PowerMAG View Navigation’s inverse normalization to transfer the coordinates from standard to individual brain space. In another session, we targeted the vertex (interhemispheric cleft, corresponding to Cz in the 10–20 system) as a control stimulation site as previously done in several other studies (Kaminski et al., 2011; Kiyonaga et al., 2014; Ritterband-Rosenbaum et al., 2014; Coutlee et al., 2016; Hill et al., 2017; Silvanto et al., 2017; Agnew et al., 2018; Koen et al., 2018; Wittkuhn et al., 2018) in order to ensure the same auditory and tactile sensations during both stimulation sessions.

We used an offline low-frequency rTMS protocol (1 Hz, 20 min, 1,200 pulses in total) with pulses delivered with an intensity of 100% of the individual motor threshold (38–57% maximum stimulator output) in order to change cortical excitability (Pascual-Leone et al., 1998) for the duration of the fMRI session. Ear plugs were used during stimulation. The time between stimulation and the start of the scanner session was kept as short as possible, to ensure that the task fMRI measurement (duration: 8 min) was completed in that time window of 15 min after stimulation. The delay between stimulation and the beginning of fMRI did not significantly differ between sessions with AG (3 min, 46 s) and vertex stimulation (3 min, 54 s), *t*(19) < 0.8. As outlined above, we compared activation changes in the early phase of the experiment – approximately 4–8 min following TMS – and in the late phase – approximately 8–12 min following TMS.

### Imaging Procedure

MRI data was acquired on a Siemens 3T whole body Trio System (Erlangen, Germany) equipped with a 32 channel head coil. Ear plugs were used to dampen scanner noise. Functional images were acquired using a gradient echo planar sequence (TR = 2,000 ms, TE = 30 ms, flip angle = 78°). Each volume contained 32 axial slices (4 mm, 20% gap) measured in ascending order with an in-plane resolution of 4 × 4 mm^2^. Following functional imaging, structural images were acquired using a T1-weighted sequence (TR = 1,900 ms, TE = 2.26 ms, TI = 900 ms, flip angle = 9°) with a resolution of 1 mm × 1 mm × 1 mm. Additionally, we measured field maps in both fMRI sessions.

### Data Analysis

#### Behavioral Data

Behavioral data was analyzed using SPSS (IBM SPSS statistics V27, IBM, Armonk, NY, United States). Response times (RTs) and error rates were computed separately for compatible and incompatible trials following AG and vertex stimulation. In order to analyze RTs and error rates in the on-task block, we conducted repeated measures ANOVAs with the factors stimulation (AG vs. vertex), compatibility (compatible vs. incompatible trials) and time (early vs. late).

#### FMRI Data

Data analysis was performed using SPM12 (Wellcome Department of Cognitive Neurology, Institute of Neurology, London, United Kingdom) based on MATLAB R2016b. As a first step during preprocessing, functional images were slice time corrected. The first 3 volumes (corresponding to 6 s) were discarded to allow for T1-equilibration effects. After that, to correct for head motion, participants’ functional images were spatially realigned and unwarped using the acquired field maps to improve the signal-to-noise ratio (Cusack and Papadakis, 2002). T1 structural images were co-registered to mean functional images and segmented into cerebrospinal fluid, white and gray matter. Images were normalized into MNI space with a spatial resolution of 3 × 3 × 3 mm^3^. Finally, images were spatially smoothed with a Gaussian kernel of 8 mm full width at half maximum to increase signal-to-noise ratio.

The experimental conditions were modeled as follows: on-task trials were modeled as events, while off-task blocks were modeled as blocks (duration 30 s). For first-level analyses we included 12 regressors of interest covering condition (on-task vs. off-task), time (early vs. late phase in the on-task/off-task part of each fMRI session) and stimulation (AG vs. vertex). In order to explicitly probe for potential compatibility-related effects, the on-task condition comprised separate regressors for compatible and incompatible trials. Block instructions and error trials of early and late phase for both sessions were modeled as regressors of no interest. All regressors were convolved with the SPM canonical hemodynamic response function with a high pass filter set to 1/128 Hz. Contrasts were created combining regressors of interest (e.g., interaction of stimulation × condition × time: AG vs. vertex, on-task vs. off-task, early vs. late phase). Activation changes were then assessed on the group level using one-sample *t*-tests (main effect: stimulation, interaction effects: stimulation × condition, stimulation × time, stimulation × time × condition) using the first level contrast images of each participant as input. For whole brain analyses, we corrected for multiple comparisons at the cluster level (FWE, *p* < 0.05), using an initial voxel-wise threshold of *p* < 0.001.

**FIGURE 2:**
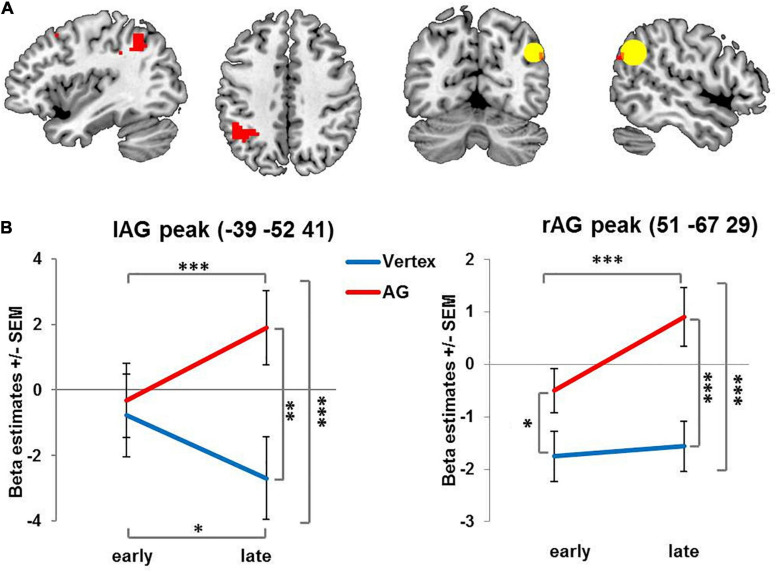
Activation changes over time: Brain regions showing increasing condition-unspecific activity over time after rTMS administered to the right AG compared to vertex. **(A)** For visualization purposes all images are thresholded at voxel level *p* = 0.001 uncorrected with red color denoting suprathreshold activation. The ROI centered on the stimulation site in right angular gyrus is indicated by the yellow circle. **(B)** Left and right AG peak coordinates for interaction of stimulation and time. AG: angular gyrus. SEM: standard error of the mean ^∗^ denotes *p* < 0.05, ^∗∗^ denotes *p* < 0.01 ^∗∗∗^ denotes *p* < 0.005.

**FIGURE 3:**
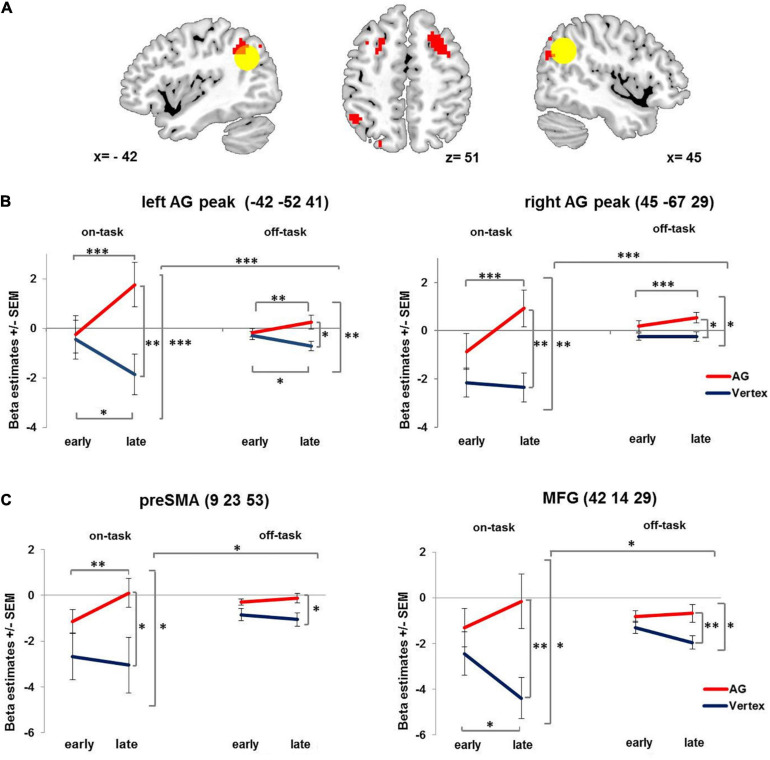
Condition-related activation changes over time. **(A)** Brain regions showing increasing condition-related activity over time after 1 Hz rTMS applied to the right AG compared to vertex. Yellow circle denotes left and right angular gyrus ROIs (12 mm). For visualization purposes all images are thresholded at voxel level *p* = 0.001 uncorrected. **(B)** Interaction of stimulation × time × condition displayed at the left and right AG peak coordinates. **(C)** Interaction of stimulation × time × condition, displayed at preSMA and MFG peaks as identified for showing a compatibility effect. AG: angular gyrus. MFG: middle frontal gyrus. PreSMA: pre-supplementary motor area. SEM: standard error of the mean. ^∗^ denotes *p* < 0.05, ^∗∗^ denotes *p* < 0.01, ^∗∗∗^ denotes *p* < 0.005.

Several previous studies (O’Shea et al., 2007; Heinen et al., 2011; Plow et al., 2014; Petitet et al., 2015; Battelli et al., 2017) reported stimulation effects in the contralateral homologous area. Based on this and the outlined literature on compensatory reorganization (Lee et al., 2003; O’Shea et al., 2007; Hartwigsen et al., 2017), TMS-induced reorganization would specifically be expected at the stimulation site and at the contralateral homologous region which were therefore defined as regions of interest. We created two spherical ROIs with a radius of 12 mm centered on the stimulated and the mirrored contralateral AG (−/ + 45 −58 33) and applied small volume correction (SVC) when accounting for multiple comparisons (see Figures 2, 3) in these regions of interest. For follow-up analyses, BOLD signals were extracted (1) for the stimulated coordinate and its contralateral homolog, (2) for the 12 mm radius ROIs centering on these left and right AG coordinates as well as, and (3) for the peak voxel of the specified contrast. Beta estimates were then analyzed in repeated measures ANOVAs with the factors stimulation (AG vs. vertex), condition (on-task vs. off-task) and time (early vs. late phase). Significant interactions were followed up by one-tailed paired *t*-tests. To foreshadow the results: The activation pattern was qualitatively similar for all three analyses, i.e., the reported results were not dependent on the specific voxel or set of voxels. For illustration, mean BOLD signals were extracted from the specified peak coordinate, if not stated otherwise.

## Results

### Stimulation Effects on Behavior

Behavioral data was analyzed as a manipulation check to ensure that participants performed the task as expected.

As noted above, one participant was excluded due to an exceptionally high error rate (32%), which was more than 3 SD above the group average (*M* = 5.6%, SD = 6%). Response omissions (0.85% of trials) were excluded from analysis. For RT analysis error trials were excluded (5.8%).

As expected, participants needed more time, and made numerically more mistakes in incompatible compared to compatible trials (RTs: M_incomp_ = 480 ms, M_comp_ = 465 ms; errors: M_incom__p_ = 5.6%, M_comp_ = 4.2%). This was reflected in a significant compatibility effect on RTs (Simon effect: *F*_1_,_19_ = 11.03, *p* = 0.005, η^2^ = 0.356), while the effect failed to reach significance for error rates (*F*_1_,_19_ = 1.98, *p* = 0.17). Importantly, there was no modulatory effect of stimulation on compatibility effects, i.e., the compatibility effects on RTs and error rates were not significantly influenced by stimulation (RTs: *F*_1_,_19_ < 0.06, error rates: *F*_1_,_19_ < 1.1). Bayesian paired sample *t*-tests (JASP Team, 2018) revealed substantial (RTs: BF_01_ = 4.1 ± 0.022) and anecdotal (errors: BF_01_ = 1.9 ± 0.01) evidence for the H0 regarding the interaction of stimulation and compatibility (according to Jarosz and Wiley, 2014). Furthermore, we found no overall stimulation effects on behavioral data, neither for RTs (*F*_1_,_19_ < 0.3, BF_01_ = 4.06 ± 0.91) nor for error rates (*F*_1_,_19_ < 2.3, BF_01_ = 2.4 ± 0.74) and no interaction involving stimulation (RTs: *F*_1_,_19_ < 2, BF_01_ = 4 ± 0.022, error rates: *F*_1_,_19_ < 1.3, BF_01_ = 2.7 ± 0.017). Additionally, there was no significant effect of time (RTs: *F*_1_,_19_ = 4.28, *p* = 0.053; errors: *F* < 1) or interaction with time (Fs < 1.95, *ps* > 0.179).

### Stimulation Effects on Brain Activity

When collapsing across early and late phases, there was neither an overall effect of stimulation on brain activity, nor an interaction of stimulation and task condition. Importantly, however, we found a significant interaction of stimulation and time in several brain regions (see Figure 2A and Table 1). Specifically, following rTMS applied over right AG but not vertex, activity in the right AG and contralateral left AG increased over time. This interaction was mainly driven by a stimulation effect in the later phase. In fact, for the left AG, initially, activity did not differ between AG and vertex stimulation. In the later phase of the experiment, left and right AG activation significantly increased after right AG rTMS compared to the early phase and compared to vertex stimulation (see Figure 2B and Table 1 for statistical results). A similar pattern emerged in the adjoining regions in the left superior parietal lobe and left supramarginal gyrus in the late phase of the experiment compared to vertex stimulation (see Table 1). The activity increase in the left AG and adjoining region was significant on the whole-brain level, whereas right AG results were based on small volume correction ROI of the stimulation site.

**TABLE 1:** Stimulation effects on brain activity.

Region	MNI coordinates			T_MAX_	Cluster size (number of voxels)
**Stimulation** × **Time**					
***AG* > *vertex late* > *early***					
Left angular gyrus	−39	−52	41	5.31	134
	−48	−52	44	5.16	
Left supramarginal gyrus	−39	−37	35	3.96	
Right angular gyrus	51	−67	29	4.37 ^SVC^	2
***AG* > *vertex (late)***					
Left superior parietal lobe	−27	−49	41	5.13	85
	−33	−49	47	4.38	
Left supramarginal gyrus	−48	−43	47	4.64	
**Stimulation** × **Time** × **Condition**					
***AG vs. vertex late vs. early on-task vs. off-task***					
Left angular gyrus	−42	−52	41	4.5 ^SVC^	12
Right angular gyrus	45	−67	29	4.3 ^SVC^	5
***AG vs. vertex late vs. early on-task***					
Left angular gyrus	−42	−55	41	5.64	149
Left superior parietal lobe	−27	−49	38	4.2	
Left supramarginal gyrus	−39	−37	35	4	
Right angular gyrus	51	−67	29	4.7 ^SVC^	3
***AG vs. vertex late vs. early off-task***					
Left angular gyrus	−48	−52	41	3.7 ^SVC^	7
Left supramarginal gyrus	−45	−49	38	3.8 ^SVC^	

*Reported brain regions were whole-brain corrected for multiple comparisons using an initial voxel-wise threshold of *p* < 0.001 and FWE cluster-level correction p_*FWE*_ < 0.05. SVC denotes peak level significant after small volume correction for the 12 mm radius ROIs centered around the stimulation site and its contralateral homologous (−/ + 45 −58 33).*

This effect was further qualified by a three-way interaction involving condition – yielding further insight into the activation pattern. In particular, the stimulation induced activation change over time in both right AG and left AG (see Figure 3 and Table 1) was especially pronounced in the on-task condition as compared to the off-task condition. The pattern of this three-way interaction was independent of the specific voxel of left and right AG from which the beta estimates were extracted. Comparable results were obtained when performing the analyses after extracting the mean beta estimate for the whole 12 mm ROI centered on the stimulation coordinate and the contralateral region as well as for the stimulation coordinate itself (see Supplementary Material).

Since the delayed activation increase after AG stimulation was more pronounced for the on-task condition, we also probed whether AG stimulation differentially affected compatible and incompatible trials. We observed a main effect of compatibility in the right AG (48 −49 41, *T* = 6.44, p_FWE_ < 0.001, cluster size = 170), the pre-supplementary motor area (9 23 53, *T* = 5.5, p_FWE_ < 0.012, cluster size = 83) and the right middle frontal gyrus in posterior dorsolateral prefrontal cortex (42 14 29, *T* = 4.9, p_FWE_ < 0.03, cluster size = 67). However, there was no whole brain interaction of stimulation and compatibility. In order to test for differential effects of stimulation and time on compatible and incompatible on-task trials, we extracted beta estimates of three regions showing a main effect of compatibility (collapsed across all voxels within each ROI with 12 mm radius) and performed repeated measures ANOVAs with the factors stimulation (AG vs. vertex), time (early vs. late phase) and compatibility (compatible vs. incompatible trials). The analyses revealed that for all three regions compatibility did not interact with stimulation (*Fs*_1_,_19_ < 2.9, *ps* > 0.103) or stimulation and time (*Fs*_1_,_19_ < 1). However, all three regions displayed the same interaction of stimulation × time × condition (independent of compatibility, *Fs*_1_,_19_ > 4.59, *ps* < 0.045) as observed for left and right AG (see Figure 3C).

## Discussion

The present study investigated the neural after-effects of 1 Hz rTMS by using fMRI to probe whether stimulation of the right AG of the IPL resulted in a condition-related and dynamic, i.e., time-dependent, functional reorganization in terms of shifted activity from stimulated region to other unaffected brain areas (Hartwigsen, 2018).

Administering a 20 min train of 1 Hz rTMS over right IPL did not lead to behavioral impairments, which is in line with previous behavioral null results after low-frequency rTMS administered to IPL regions (Rossi et al., 2006; Riemer et al., 2016). Typically, the effect of 1 Hz rTMS is expected to inhibit cortical excitability and perturb function beyond stimulation for roughly as long as the duration of the stimulation (Wassermann et al., 1996; Chen et al., 1997; Boroojerdi et al., 2000; Muellbacher et al., 2000; Lewald et al., 2002). However, some studies reported shorter after-effects on behavior (Robertson et al., 2003; O’Shea et al., 2007; Eisenegger et al., 2008; Plow et al., 2014; Battelli et al., 2017) and additional evidence for rapid reorganization on the brain level (Lee et al., 2003; O’Shea et al., 2007; Plow et al., 2014; Battelli et al., 2017).

Supporting the latter notion, in the present study we did not find overall changes in brain activity following 1 Hz rTMS applied to the right AG. Instead, we observed condition-related dynamically changed activity of both the unstimulated contralateral region and the stimulated region itself. Specifically, in the later phase of the experiment (approximately 8 – 12 min after stimulation) bilateral AG activity was increased after rTMS applied to the right AG relative to both an earlier phase after right AG stimulation as well as relative to vertex stimulation. This was most pronounced during the on-task condition. Although a qualitatively similar effect was observed for the off-task condition, delayed activation increase was significantly stronger when active task performance was required. This is in accordance with our hypothesis that rTMS after-effects are condition-related. Interestingly, a similar pattern of delayed activation increase following AG stimulation was also found in other brain regions of a task-positive network related to executive functions (pre-SMA and posterior DLPFC in the right hemisphere).

In the following section we will briefly discuss how these dynamic changes suggest mechanisms of rapid functional reorganization that might compensate for focal disruption. After that, we elaborate on how rapid reorganization may explain inconsistencies in the literature like absent activation or connectivity changes at the stimulated brain area and behavioral null results.

### Rapid Functional Reorganization Mechanisms

First, our pattern of condition-related rTMS after-effects and the absence of an overall stimulation effect on the stimulated area suggests resilience or robustness, described as an “up-regulation of task-related activity to maintain task processing” (Hartwigsen, 2018). Following vertex stimulation, the right AG was more strongly engaged in the off-task condition as compared to the on-task condition (see Figure 3B) a pattern typically observed in brain regions constituting the DMN (Buckner et al., 2008). Following AG stimulation, activity of the stimulated right AG was upregulated in the late phase of the session compared to the early phase, especially in the on-task condition. Furthermore, it was equally engaged in the on-task as in the off-task condition (Figure 3B and Supplementary Figure 1B), suggesting that the AG became part of a task-positive network. This “up-regulation of task-related activity” (Hartwigsen, 2018) accompanied by unchanged performance may hint at a compensatory effect.

Secondly, the delayed activity increase in the contralateral left AG following right AG stimulation relates to another possible reorganization mechanism, the “recruitment of homologous regions” (Hartwigsen, 2018). Again, the pattern of increased activity in the late phase of the session was especially pronounced in the on-task condition. The increased involvement of the contralateral brain region corroborates previous findings on changed activity and connectivity patterns in the homolog of the stimulated brain region after rTMS (O’Shea et al., 2007; Grefkes et al., 2010; Hartwigsen et al., 2013; Plow et al., 2014; Petitet et al., 2015; Balan et al., 2017; Battelli et al., 2017). O’Shea et al. (2007) found compensatory reorganization in terms of increased activity of the contralateral premotor cortex after offline stimulation. Similarly, Plow et al. (2014) reported behavioral compensation by activity increase in the unstimulated left parietal cortex 5–12 min after right parietal cortex stimulation. The activity changes were accompanied by initially weakened functional connectivity, followed by a recovery to undisturbed connectivity levels and a delayed strengthening of functional connectivity between homologous regions in both hemispheres (Battelli et al., 2017). In accordance with the present study these findings illustrate the rapid reorganization of contralateral homologous brain activation after focal perturbation (Plow et al., 2014; Battelli et al., 2017).

Finally, several authors found remote network effects after focal perturbation (Ruff et al., 2006; Sack et al., 2007; de Vries et al., 2009; Hartwigsen et al., 2017; Croce et al., 2018) constituting another reorganization mechanism referred to as “compensation within and between networks” (Hartwigsen, 2018). Here, we also report evidence for network effects following rTMS applied to the AG. More specifically, activity of the pre-SMA and the posterior DLPFC showed stronger activation in the late phase following AG rTMS especially for on-task condition. These regions have been shown to be crucially involved in action planning, attentional control, managing response conflict and behavioral inhibition (Botvinick et al., 2001; Mostofsky and Simmonds, 2008; Brass et al., 2009; Shackman et al., 2009; Cieslik et al., 2015; Power et al., 2015; Igelström and Graziano, 2017) – functions and processes necessary to successfully perform in a spatial Simon task as used in our on-task condition (Liu et al., 2004; Olk et al., 2015; Cespón et al., 2020). Thus, it is conceivable that pre-SMA and posterior DLPFC as part of a task-positive network could also act in a compensatory manner. In fact, according to several studies the AG is part of a network hub connected with the task-negative DMN (Buckner et al., 2008; Hagmann et al., 2008; Vincent et al., 2008; Igelström and Graziano, 2017) and task-positive networks such as the fronto-parietal control network (Vincent et al., 2008; Igelström and Graziano, 2017; Dixon et al., 2018). More specifically, it has been suggested that the IPL constitutes an adaptive task-control hub of the fronto-parietal control network that can flexibly change its functional connectivity with multiple brain networks across different conditions (Cole et al., 2013). Functional reallocation of the IPL from the DMN to the fronto-parietal control network may explain remote effects in different networks and the pattern of up-regulated task-related activity in the later phase following AG stimulation and could be part of a compensatory mechanism after focal disruption. Together, such rapid reorganization mechanisms might explain some contradictory results of different TMS studies – as we will elaborate in the next section.

### Rapid Functional Reorganization May Explain Inconsistent Findings in Stimulation Literature

Previous studies using low-frequency stimulation protocols taken to be inhibitory yielded inconsistent results showing either increased or decreased cortical excitability and functional connectivity with local and remote brain regions (Pascual-Leone et al., 1998; Nahas et al., 2001; Eisenegger et al., 2008; Eldaief et al., 2011; Beynel et al., 2020; Castrillon et al., 2020). According to Castrillon et al. (2020), this discrepancy between decreased and increased connectivity/excitability might indeed be explained by the brain area which is stimulated: early sensory areas showed decreased resting state connectivity with remote areas while higher cognitive areas showed increased resting state connectivity after low-frequency rTMS. In the present study, the IPL, a flexible hub to various networks was investigated and local and remote rTMS after-effects were found to be more pronounced when participants actively performed a task. This particularly fits previous results by Lee et al. (2003) who found increased activity in the stimulated area (suggesting resilience) and an additional condition-related, in this case, movement-related, activity increase in the contralateral area (suggesting within-network reorganization). In line with these findings, the present study supports the notion that the direction of activity change following stimulation is influenced by the functional state a particular brain region is in i.e., its current involvement in a task. Together, these findings hint towards more complex, condition-related reorganization that might explain the contradictory results of increased and decreased cortical excitability after low-frequency rTMS.

Furthermore, the temporal dynamics of functional reorganization such as those observed in the present study might also explain mixed findings in the TMS literature. Some previous studies did not report significant activation changes in stimulated brain areas, but instead found changes in remote brain regions (Bohning et al., 1999; Baudewig et al., 2001; Bestmann et al., 2004, 2005; O’Shea et al., 2007; Castrillon et al., 2020). While brain activity and connectivity (as well as the corresponding behavior) might be inhibited when measured instantly after stimulation (Chen et al., 1997; Pascual-Leone et al., 1998; O’Shea et al., 2007), they might have returned to baseline level or even show a compensatory increase when fMRI measurement starts 4–5 min after the end of stimulation (Lee et al., 2003; O’Shea et al., 2007; Plow et al., 2014; Battelli et al., 2017). A pattern of unchanged or decreased activation in an early phase followed by increased activation in a later phase may thus effectively cover an overall stimulation effect, as was the case in the present study.

### Potential Limitation

A possible limitation could be that both, effective (AG) and control (vertex) stimulation were performed on the same day due to feasibility considerations. However, we took measures to prevent carry over effects. First, there was a time window of at least 35 min between the end of the first stimulation and the beginning of the second stimulation, based on the assumption that the effects of 1 Hz stimulation in healthy participants on behavior are generally short-lived (Rossi et al., 2020). Moreover, as outlined before, brain activation changes following 15 min 1 Hz rTMS returned to baseline after 9 min (Eisenegger et al., 2008) showcasing that neural after-effects may also be very short-lived. Second, we counterbalanced the order of stimulation across participants to rule out that the resulting stimulation effects were due to order effects.

Another potential limitation refers to the vertex stimulation chosen as control method instead of a sham or no-TMS condition. It was chosen over other control measures based on feasibility and vast literature background (Ruff et al., 2006; Kaminski et al., 2011; Kiyonaga et al., 2014; Ritterband-Rosenbaum et al., 2014; Coutlee et al., 2016; Hill et al., 2017; Silvanto and Cattaneo, 2017; Agnew et al., 2018; Koen et al., 2018; Wittkuhn et al., 2018) in order to ensure the same auditory and tactile sensations during both stimulation sessions. We cannot rule out, however, that our control condition might have also modulated the brain state of the participants. In fact, Jung et al. (2016) found widespread deactivations in areas of the DMN after inhibitory vertex stimulation in resting state conditions. However, we used a significantly lower stimulation intensity (100% resting motor threshold compared to 120%). Most importantly, we show that our results are driven by upregulation of activation over time after rTMS of the AG, specifically under on-task conditions, rather than by systematic changes following rTMS of the vertex. Therefore, the main results of this study, the condition-related and time-dependent reorganization after focal perturbation do not hinge on the specific control stimulation method.

## Conclusion

In summary, our combined rTMS-fMRI study provides further evidence for a rapid functional reorganization of the brain following low-frequency stimulation. Specifically, 1 Hz rTMS applied over the right IPL led to increased activity in both left and right IPL in the late phase after stimulation, which was more pronounced in an on-task condition requiring active task performance. Thus, stimulation after-effects were condition-related and dynamic in being time-dependent. The reported dynamic changes following IPL stimulation are in line with recently proposed rapid reorganization mechanisms after focal disruption, i.e., resilience, recruitment of homologous regions and inter- and intra-network compensation (see Hartwigsen, 2018). The dynamic pattern of functional reorganization may explain inconsistencies in the TMS literature such as contradictory results after low-frequency stimulation and may cover overall stimulation effects by opposite after-effects in early and later phases after stimulation. Notwithstanding that, the exact mechanisms of functional reorganization following rTMS to different brain regions are as of yet not fully understood. Importantly, rapid reorganization after rTMS poses a challenge for scientific and clinical application exemplified in behavioral null results and response failures. Therefore, further combined and concurrent rTMS-fMRI studies are needed to systematically investigate the complex interplay of different brain systems under different conditions to close the explanatory gap between brain function and behavior.

## Data Availability Statement

The raw data supporting the conclusions of this article will be made available by the authors, without undue reservation.

## Ethics Statement

The studies involving human participants were reviewed and approved by the Ethics Committee of the Technische Universität Dresden. The participants provided their written informed consent to participate in this study.

## Author Contributions

JJ, KZ, HR, and UW designed the study. JJ collected, analyzed the data, and wrote original draft. JJ, KZ, HR, FK, and UW wrote, reviewed, edited, and approved the final manuscript. HR and UW were project administrators. UW, HR, and KZ supervised JJ. All authors contributed to the article and approved the submitted version.

## Conflict of Interest

The authors declare that the research was conducted in the absence of any commercial or financial relationships that could be construed as a potential conflict of interest.

## Publisher’s Note

All claims expressed in this article are solely those of the authors and do not necessarily represent those of their affiliated organizations, or those of the publisher, the editors and the reviewers. Any product that may be evaluated in this article, or claim that may be made by its manufacturer, is not guaranteed or endorsed by the publisher.

## Footnotes

1 Importantly, we do not refer to the dependency of stimulation effects on the initial brain state during stimulation called state-dependency of TMS effects (Silvanto and Cattaneo, 2017; Silvanto et al., 2017). We refer to the post-stimulation brain states which differ depending on task condition thereby potentially altering TMS after-effects (condition-related reorganization).
