# Supplementary material for: Low-Frequency TMS Results in Condition-Related Dynamic Activation Changes of Stimulated and Contralateral Inferior Parietal Lobule
Source: Front Hum Neurosci. 2021 Jul 23;15:684367. doi: 10.3389/fnhum.2021.684367 (PMC8342925; doi:10.3389/fnhum.2021.684367)
Supplement: Supplementary file 1 [file Data_Sheet_1.docx]

Supplementary Material

# Stimulation effects on activation

*
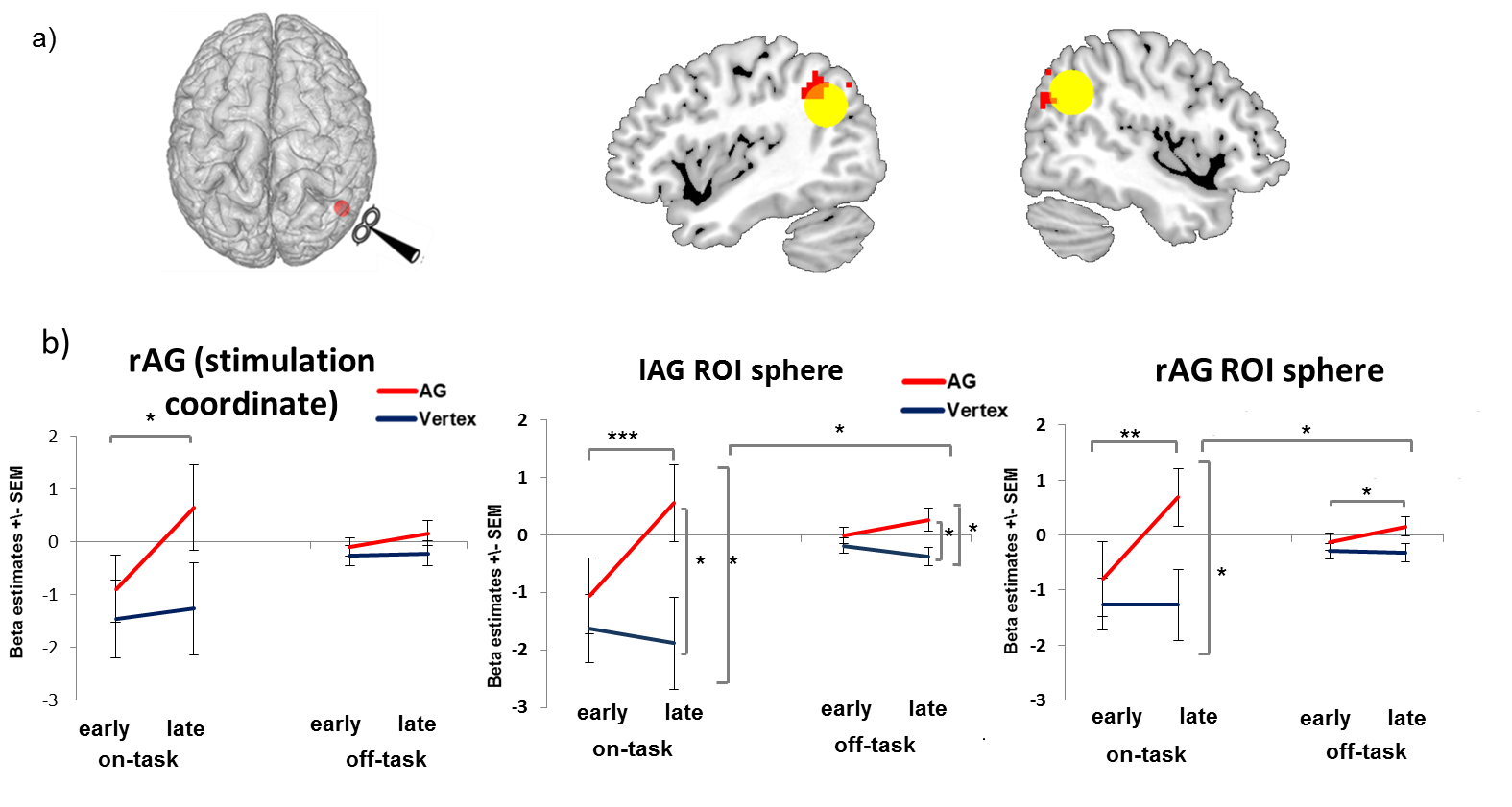
*

**Fig. S1 Condition-related activity increase over time.** a) Brain regions showing increasing condition-related activity over time after rTMS administered to the right AG compared to vertex. Red circle denotes stimulation coordinate (45 -58 33). Yellow circle denotes left and right angular gyrus ROIs (12 mm). For visualization purposes all images are thresholded at voxel level p=.001 uncorrected. b) Right stimulation coordinate and mean activation in both ROIs show the same stimulation x condition x time pattern. AG: angular gyrus. SEM: standard error of the mean * denotes p<.05, ** denotes p<.01, *** denotes p<.005

# Resting state analysis

## Resting State fMRI

Resting state fMRI started approximately 11-15 minutes after stimulation. For resting state measurement, participants were told to keep eyes open and fixated on a cross. Patriat et al. (2013) showed significantly higher reliability and network consistency for resting state measurements with eyes fixated compared to open or closed eyes (Patriat et al., 2013). For imaging a multilevel band was used with factor 6 (TR=987ms , TE=32.6 flip angle = 62◦). Voxel size of the 72 slices were 2mm x 2mm x 2mm.

## Resting state data

DPARSF (Data Processing assistant for resting state fMRI) was used for preprocessing of resting state data and functional connectivity analysis of resting state data (Yan et al. 2016). Images were transformed to nifty, realigned to T1 with manual quality control, segmented into gray matter, white matter and CSF and normalized by using DARTEL. Motion correction was done by the higher order Friston 24-parameter model (Friston et al., 1999). Signals from WM and CSF were regressed out to reduce effects of respiration and heartbeat. Temporal filtering was set to (0.01–0.1 Hz). Resting state analysis was conducted as DPABI seed based correlation analysis. Connectivity with AG ROI (54 -58 33, radius 6) was analyzed based on temporal correlation of activation. The smoothed connectivity maps for each participant and session (vertex and AG session separately) were analyzed in DPABI and in SPM with paired t-tests.

## Resting state results

Paired t-test of the functional connectivity (independent of DPABI or SPM analysis) showed increased coupling of the right AG and the temporal pole extending into the right central operculum after AG stimulation. We report the SPM results (SPM: FWE full brain corrected p_FWE_<.02 in blue). There were no changes for resting state connectivity in the contralateral left angular gyrus.


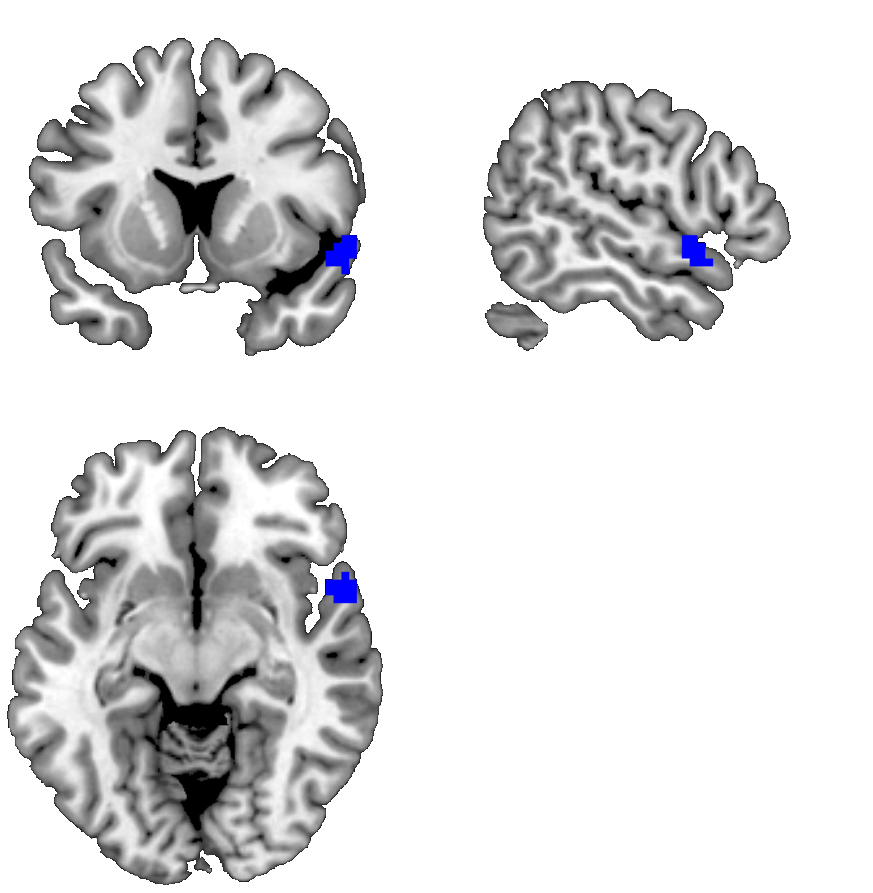
**y=12**

**Fig. S2 Resting state connectivity change** of AG and temporal pole after AG TMS compared to vertex TMS. Image is thresholded at voxel level p=.001 and FWE whole brain corrected p_FWE_<.05 .

Table S1: Resting state results

| Resting state AG > Vertex | | |  |  | Cluster size |
| --- | --- | --- | --- | --- | --- |
| R Temporal Pole  R central operculum | 54  42 | 12  9 | 96 | T=5.2 p=.019  T=4.8 | 106 |
|  |  |  |  |  |  |

Further subtle differences in resting state connectivity between the vertex and the AG session may represent the fading out of stimulation effects or after-effects of the before conducted task.

**Additional references for supplementary material**

Friston, K.J., Holmes, A.P., Price, C.J., Büchel, C., Worsley, K.J., 1999. Multisubject fMRI studies and conjunction analyses. Neuroimage 10, 385–396. https://doi.org/10.1006/nimg.1999.0484

Patriat, R., Molloy, E.K., Meier, T.B., Kirk, G.R., Nair, V.A., Meyerand, M.E., Prabhakaran, V., Birn, R.M., 2013. The effect of resting condition on resting-state fMRI reliability and consistency: A comparison between resting with eyes open, closed, and fixated. Neuroimage 78, 463–473. https://doi.org/10.1016/j.neuroimage.2013.04.013

Yan, C.G., Wang, X. Di, Zuo, X.N., Zang, Y.F., 2016. DPABI: Data Processing & Analysis for (Resting-State) Brain Imaging. Neuroinformatics 14, 339–351. https://doi.org/10.1007/s12021-016-9299-4
